# Recurrence of cervical intraepithelial neoplasia in human immunodeficiency virus-infected women treated by means of electrosurgical excision of the transformation zone (LLETZ) in Rio de Janeiro, Brazil

**DOI:** 10.1590/1516-3180.2013.1316578

**Published:** 2013-12-01

**Authors:** Fábio Russomano, Bruno Reis Paz, Maria José de Camargo, Beatriz Gilda Jegerhorn Grinstejn, Ruth Khalili Friedman, Maria Aparecida Pereira Tristao, Caroline Alves Oliveira

**Affiliations:** I MD, PhD. Deputy Director, Department of Education, and Head, Colposcopy Sector, Instituto Nacional de Saúde da Mulher, da Criança e do Adolescente Fernandes Figueira/Fundação Oswaldo Cruz (IFF/Fiocruz), Rio de Janeiro, Brazil.; II Medical Student, Universidade Federal do Rio de Janeiro (UFRJ), Rio de Janeiro, Brazil.; III MD, PhD. Gynecologist, Department of Gynecology. IFF/Fiocruz, Rio de Janeiro, Brazil.; IV MD, PhD. Infectologist, Clinical Trials Unit, Instituto de Pesquisa Clínica Evandro Chagas (IPEC)/Fundação Oswaldo Cruz (Fiocruz), Rio de Janeiro, Brazil.; V MD, PhD. Pathologist, Department of Pathological Anatomy, Instituto Fernandes Figueira (IFF)/Fundação Oswaldo Cruz (Fiocruz), Rio de Janeiro, Brazil.; VI MD, MSc. Obstetrician and Gynecologist, Hospital Federal de Bonsucesso, Rio de Janeiro, Brazil.

**Keywords:** Cervical intraepithelial neoplasia, Electrosurgery, Recurrence, Cohort studies, HIV, Neoplasia intra-epitelial cervical, Eletrocirurgia. Recidiva, Estudos de coortes, HIV

## Abstract

**CONTEXT AND OBJECTIVE:**

Human immunodeficiency virus (HIV)-infected women have higher incidence, prevalence, persistence and recurrence of pre-invasive cervical lesions (CIN II or III). The aim here was to investigate the risk of recurrence of CIN II/III among HIV-infected women (HIV+) and uninfected women in a cohort treated by means of large-loop excision of the transformation zone (LLETZ).

**DESIGN AND SETTING:**

Cohort study conducted at Instituto Fernandes Figueira/Fundação Oswaldo Cruz (IFF/Fiocruz).

**METHODS:**

60 HIV+ and 209 HIV-negative patients were included in a cohort for follow-up after undergoing LLETZ to treat CIN II/III. A histopathological diagnosis of CIN II/III during the follow-up was taken to constitute recurrence. The following possible confounding variables were assessed: age at treatment and at end of follow-up; histological grade of intraepithelial disease treated; surgical margin involvement; adequacy of colposcopy during the follow-up; CD4+ lymphocyte count; HIV viral load; and type of antiretroviral therapy.

**RESULTS:**

Among the 60 HIV+ women, six showed recurrent disease during the follow-up. However, among the 209 HIV-negative women, seven showed a new precursor lesion. The relative risk of disease recurrence in the HIV+ women was 4.21 (95% CI = 1.42 to 12.43). The Kaplan-Meyer curve showed that the risk of recurrence was significantly higher among HIV+ women (log-rank test: P = 0.0111).

**CONCLUSION:**

The HIV+ women in our cohort presented a risk of CIN II/III recurrence at least 42% higher than among the HIV-negative women. These patients should form part of a rigorous screening and follow-up protocol for identification and appropriate treatment of cervical cancer precursor lesions.

## Abstract (pt)

**CONTEXTO E OBJETIVOS:**

Mulheres infectadas pelo vírus da imunodeficiência humana (HIV) apresentam maior incidência, prevalência, persistência e recorrência após tratamentos de lesões pré-invasivas do colo uterino (NIC II ou III). O objetivo foi verificar o risco de recorrência de NIC II/III em mulheres infectadas pelo HIV (HIV+) e não infectadas (HIV-) em uma coorte tratada pela exérese eletrocirúrgica da zona de transformação do colo uterino (EZT).

**TIPO DE ESTUDO E LOCAL:**

Estudo de tipo coorte realizado no Instituto Fernandes Figueira/Fundação Oswaldo Cruz (IFF/Fiocruz).

**MÉTODOS:**

60 HIV+ e 209 HIV- foram incluídas em uma coorte após terem sido submetidas à EZT para tratamento de NIC II/III. Foi considerado como recorrência o diagnóstico histopatológico de NIC II/III. Foram estudadas as seguintes variáveis possivelmente confundidoras: idade no tratamento e ao final do seguimento, grau histológico da doença intra-epitelial tratada, comprometimento de margens, adequação da colposcopia no seguimento, contagem de linfócitos CD4+, carga viral de HIV e tipo de terapia antiretroviral.

**RESULTADOS:**

Dentre as 60 mulheres HIV+, 6 apresentaram doença recorrente durante o seguimento. De 209 HIV-, 7 apresentaram uma nova lesão precursora. O risco relativo de recorrência de doença nas HIV+ foi de 4,21 (IC 95% 1,42-12,43). Uma curva de Kaplan-Meyer mostra que o risco de recorrência é significativamente maior em mulheres HIV+ (teste de *log-rank*: P = 0,0111).

**CONCLUSÃO:**

Mulheres HIV+ em nossa coorte apresentaram risco de recorrência pelo menos 42% maior do que mulheres HIV-. Essas pacientes devem fazer parte de um protocolo de rastreio e acompanhamento rigoroso para identificação e tratamento adequado das lesões precursoras do câncer de colo uterino.

## INTRODUCTION

The epidemic caused by the human immunodeficiency virus (HIV) infection has spread throughout the world. However, the survival of HIV-infected patients has increased significantly through improvement of its clinical management and with the advent of potent antiretroviral therapy (highly active antiretroviral therapy, HAART).^1^ ^,^ ^2^ Consequently, these patients have become a target for chronic and degenerative diseases. Among these diseases, increased frequency of cervical cancer precursors, i.e. cervical intraepithelial neoplasia II and III (CIN II/III), has been observed. These precursors take on special characteristics among HIV-infected women.

HIV-infected women, especially those with low levels of CD4+ cells, present higher risk of persistent infection by HPV (human papillomavirus) and higher rates of low and high-grade squamous intraepithelial lesions and invasive carcinoma of the uterine cervix, in comparison with HIV-negative women. Because of this relationship, presence of CIN is considered to be a condition related to HIV infection, whereas invasive carcinoma of the cervix is an AIDS-defining illness.^3^

Cervical cancer is still a health problem in Brazil, since it is the third most frequent tumor in women, according to 2012 estimates from the National Cancer Institute (Instituto National de Cancer, INCA),^4^ and it accounts for the deaths of 230,000 women per year.^5^

There is broad evidence that treating CIN, especially high-grade cases (CIN II and III), is an effective measure for preventing cervical cancer. Several studies have shown that HIV+ women present increased risk of persistence or recurrence of CIN after treatment,^6^ which is usually related to immunodepression and low levels of CD4+ T lymphocytes,^7^ as well as to the extent of endocervical CIN.^3^ It seems that CD3 T lymphocytes may participate in recurrence of CIN III in HIV+ women who were treated by means of cervical conization.^8^

Large-loop excision of the transformation zone (LLETZ) is a first-line therapeutic method for treating high-grade CIN and, in some cases, low-grade CIN.^7^ The success rates from treating CIN with LLETZ are high, ranging from 73% to 98%. When used to treat type I or II transformation zones (completely ectocervical or partially endocervical, with the squamocolumnar junction, SCJ, seen within the first centimeter of the endocervical canal), LLETZ is comparable to cold conization and other ablative procedures.^9^ ^-^ ^11^ It presents less morbidity due to shorter duration of surgery, use of local anesthesia and lower blood loss.^12^

In an initial paper on the risk of recurrence of these diseases during the first years of follow-up of this cohort, we demonstrated that the CIN II/III recurrence rate was 30.06/10,000 women per month among HIV-positive women, and of 4.88/10,000 women per month in the HIV-negative group (relative risk, RR = 6.16; 95% confidence interval, CI = 2.07-18.34).^6^ The present study shows the results from a longer follow-up and with a larger number of participants in the cohort.

## OBJECTIVES

To estimate the incidence and relative risk of recurrence of CIN II/III among HIV-infected women, after LLETZ, compared with women who were not infected by this virus.

## METHODS

### Study design

Our study was a non-concurrent (prospective and retrospective) comparative cohort study among women with and without HIV infection who underwent LLETZ to treat CIN II/III. The patients were included in the study from the time of their treatment and were monitored by means of colpocytology and colposcopy every six months.

### Inclusion criteria

Study Group: HIV+ women diagnosed with CIN II/III who were treated with LLETZ in the Cervical Pathology and Colposcopy Sector, Department of Gynecology, Instituto Nacional de Saúde da Mulher, da Criança e do Adolescente Fernandes Figueira Fundação Oswaldo Cruz (IFF/Fiocruz). These women did not have any history of treatment preceding the therapy that led to their inclusion in the cohort, for the same level of disease, and they underwent at least one cytological and colposcopic evaluation six months after treatment.

Control group: HIV-negative women diagnosed with CIN II/III, with the same characteristics, who were treated and monitored at IFF/Fiocruz. 

### Sampling, storage, and data processing

In this study, information on the study factor and outcome, and on confounding variables associated with the study factor and/or outcome that possibly influenced this relationship, was gathered.^13^ Thus, the variables measured were: presence of HIV; diagnosis of recurrent CIN II/III, which was histologically verified at any time during monitoring after LLETZ; age at treatment and at end of follow-up; histological grade of intraepithelial disease treated by LLETZ (before inclusion); surgical margin involvement in the segment excised by means of LLETZ (possible incomplete excision), as reported from the histopathological examination (before inclusion); adequacy of colposcopy during the follow-up (satisfactory or unsatisfactory); CD4+ T lymphocyte count; HIV viral load quantification; and type of antiretroviral therapy. All these variables were obtained at the time of the diagnosis/treatment and at every follow-up appointment after treatment. Pap smears were provided on these occasions, and, at the next appointment, guided by the result from this test, colposcopy was performed by one of the colposcopists in charge of the Cervical Pathology and Colposcopy Sector of IFF/Fiocruz. In the event of major colposcopic alterations, or minor ones with cytological findings suggesting CIN II or III, a biopsy was applied. When the SCJ was not completely visible and the cytology showed high grade atypia, cervical conization was indicated. Recurrence was considered to have occurred if a diagnosis of CIN II or III was observed in histological specimens obtained by colposcopically guided biopsy or other surgical method (LLETZ or conization). 

Information on the HIV-related disease was obtained from documents supplied by the patients themselves or by consulting the medical records in the units where they were being monitored for their HIV infection to be treated. The measures taken into consideration were the ones closest to the follow-up visits.

The patient losses were due to moving home to other states or, mostly among HIV patients, death due to causes other than cervical neoplasia.

The data were stored in a local database at IFF/Fiocruz. At each visit, data relating to that cervical disease and HIV infection, when applicable, were collected. The data were stored in Microsoft Access and then processed using the Epi-Info version 6.4d and Stata 10.0 statistical packages.

### Measurements applied and analysis

To estimate the incidence and relative risk of recurrence of pre-invasive lesions, we used a measurement of cumulative incidence in the first and second years and incidence density over the course of the study in each group. In this measurement method, each patient contributed proportionally over the time interval for which she was monitored. To estimate the risk of recurrence over time, we used the Kaplan-Meyer method.

The present work was submitted to and approved by the research ethics committee of IFF/Fiocruz.

## RESULTS

Two hundred and sixty-nine patients met the inclusion criteria, distributed according to Table 1. There were no significant differences concerning age during treatment or at the end of the follow-up, although the HIV-negative patients had been monitored for a significantly longer time.

**Table 1:** Characteristics of patients included in the cohort (from IFF/Fiocruz, 1996-2010) and diagnoses resulting from specimens obtained from large-loop excision of the transformation zone (LLETZ) for cervical intraepithelial neoplasia (CIN)

	HIV-positive	HIV-negative	Total	P-value
Number (%)	60 (22.3)	209 (77.7)	269	-
Mean age at time of treatment (SD)	31.6 (6.44)	32.15 (7.64)	32.03 (7.38)	0.576*
Mean age at the end of the follow-up (SD)	36.55 (6.74)	38.95 (9.00)	38.41 (8.59)	0.026*
Mean length of follow-up in years (SD)	4.93 (3.59)	6.95 (4.12)	6.50 (4.09)	< 0.001*
CIN grade				
CIN II (%)	33 (55.0)	78 (37.3)	111 (41.3)	0.011†‡
CIN III (%)	26 (43.3)	130 (62.2)	156 (58.0)	
CIN II/III§ (%)	1 (1.7)	1 (0.5)	2 (0.7)	
Involved margins				
Ectocervical				
Involved (%)	13 (21.7)	22 (10.5)	35 (13.0)	0.015†||
Free (%)	43 (71.7)	183 (87.6)	226 (84.0)	
Impaired assessment (%)	4 (6.7)	4 (1.9)	8 (3.0)	
Endocervical				
Involved (%)	14 (23.3)	32 (15.3)	46 (17.1)	0.112†||
Free (%)	43 (71.7)	174 (83.3)	217 (80.7)	
Impaired assessment (%)	3 (5.0)	3 (1.4)	6 (2.2)	
Stromal				
Involved (%)	1 (1.7)	0	1 (0.4)	0.217†||
Free (%)	56 (93.3)	206 (98.6)	262 (97.4)	
Impaired assessment (%)	3 (5.0)	3 (1.4)	6 (2.2)	
Some margin involvement	20 (33.3)	44 (21.1)	64 (23.8)	0.049†

* T test (equal variances not assumed)

† Chi-square

‡ Cases of CIN II/III excluded

§ Not possible to differentiate CIN II from III

|| Cases of impaired assessment were excluded.

This study also observed that there were significant differences in the percentages of CIN III (higher in HIV-negative women) and ectocervical margin involvement in the specimens resulting from LLETZ (higher in HIV+). This was not observed for the other margins (endocervical and stromal) (Table 1).

Among the 60 HIV+ women, 6 presented recurrent disease during the follow-up. But among the 209 HIV-negative women, 7 showed a new precursor lesion. These figures were used to calculate the incidence density, which showed that the relative risk was 4.21 (95% CI = 1.42 to 12.43). The overall incidence of recurrence was 7.43 per 1,000 women per year (Table 2).

**Table 2:** Cumulative incidence and incidence density of cervical intraepithelial neoplasia (CIN) II/III after large-loop excision of the transformation zone (LLETZ) (IFF/Fiocruz, 1996-2010)

	HIV-positive	HIV-negative	Total	P-value
Total number of women	60	209	269	-
Recurrence (%)	6 (10.0)	7 (3.3)	13 (4.8)	0.045*
Women per year	296	1453		
Incidence density (per 1,000 women per year)	20.3	4.8	7.43	0.0048†
Relative risk (95% confidence interval)	4.21 (1.42-12.43)	-	-	-
Recurrence up to the 1st year (n, %)	1 (1.7)	0 (0)	1 (0.3)	-
Recurrence up to the 2nd year (n, %)	1 (1.7)	3 (1.4)	4 (1.4)	-

* Fisher's test

†Chi-square.

**Figure 1.**
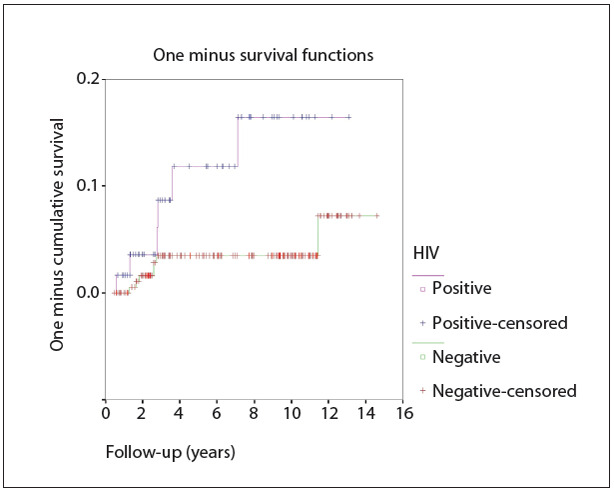
Kaplan-Meyer curve showing the probability of recurrence over the years of follow-up among women undergoing large-loop excision of the transformation zone (LLETZ) (IFF/Fiocruz, 1996-2010) to treat CIN II or III (IFF/Fiocruz, 1996-2010).

To estimate the risk of recurrence over time, we used the Kaplan-Meyer method (Figure 1). It could be seen that HIV+ women presented a significantly higher risk of CIN II/III recurrence after LLETZ (log-rank test: P = 0.0111). Because of the small number of outcomes, it was not possible to identify a period over which detection would be most likely. Moreover, it was not possible to perform analysis on subgroups of HIV+ women according to whether they were using potent antiretroviral therapy, since almost all the women were using it, or according to CD4+ T lymphocyte levels, since this information was only available for a small percentage of the women.

In an attempt to identify other possible confounding factors, we tested the associations between grade of treated disease, some margin involvement, age at the time of treatment, age at the end of the follow-up and length of follow-up until detection of recurrent disease. Only the length of follow-up was statistically associated with recurrence, but this was an inverse relationship (Table 3).

**Table 3:** Possible factors related to recurrence after large-loop excision of the transformation zone (LLETZ) (IFF/Fiocruz, 1996-2010) to treat cervical intraepithelial neoplasia (CIN)

	Presence of recurrence	Absence of recurrence	Total	P-value
CIN III (%)	5 (38.5)	151 (59.4)	156 (58.4)	0.134*
CIN II (%)	8 (61.5)	103 (40.6)	111 (41.6)	
Some margin involvement (%)	4 (30.8)	60 (23.4)	64 (23.8)	0.374†
No margin involvement (%)	9 (69.2)	196 (76.6)	205 (76.2)	
Mean age at time of treatment (SD)	31.16 (7.93)	32.07 (7.37)	32.03 (7.38)	0.665‡
Mean age at the end of the follow-up after treatment (SD)	34.51 (8.19)	38.61 (8.58)	38.41 (8.59)	0.093‡
Mean length of follow-up in years (SD)	3.26 (2.92)	6.67 (4.08)	6.50 (4.09)	0.001§

SD = standard deviation

* Chi-square

† Fisher's test

‡ T test (equal variances assumed)

§ T test (equal variances not assumed).

In another analysis, we sought to identify factors related to residual disease. Thus, we searched for an association between incompletely treated disease (some margin involvement) and persistent disease over the first two years. No such association could be demonstrated (Table 4).

**Table 4:** Analysis of margin involvement in persistent disease over first two years after large-loop excision of the transformation zone (LLETZ) (IFF/Fiocruz, 1996-2010)

	Presence of lesion within first two years after LLETZ	Absence of lesion within first two years after LLETZ	Total	P-value
Some margin involvement (%)	1 (20.0)	14 (29.2)	15 (28.3)	0.561*
No margin involvement (%)	4 (80.0)	34 (70.8)	38 (71.7)	

* Fisher's test.

## DISCUSSION

The main finding of our study is that the risk of recurrence of cervical cancer precursor lesions was 4.21 times higher among HIV+ patients. This risk was at least 42% higher than among HIV-negative women, possibly reaching up to 12 times more (95% CI = 1.42 to 12.43).

The HIV-negative patients were monitored for a significantly longer time. However, there were no significant differences concerning age during the treatment or at the end of the follow-up. 

Regarding the remaining possible confounding factors, we did not find any statistically significant differences relating to the grade of CIN treated, margin involvement, age at the time of treatment or age at the end of the follow-up. Paraskevaidis et al.^14^ reviewed all cases of LLETZ between 1989 and 2000. They found that the only feature that reached statistical significance was age, which was greater in patients with residual disease.

There was a significant difference in the percentages of CIN III and ectocervical margin of involvement in the specimens resulting from LLETZ, and this was higher in HIV-negative patients. Although these factors are related to higher risk of recurrent disease,^15^ ^-^ ^18^ it was not possible to correlate the presence of margin involvement with residual disease in our study.

In a nested case-control study, Lodi et al. found that HIV infection and glandular involvement were independently associated with CIN recurrence.^19^ However, this histopathological characteristic was not taken into consideration in our study.

In our study, only the length of the follow-up was statistically associated with recurrence. Nevertheless, this was an inverse relationship, which contradicts the hypothesis that this factor contributes towards the risk of recurrent disease. This can be explained by the higher risk of lesion persistence observed in HIV-infected patients, especially those with low levels of CD4+ cells detected in the early years of follow-up.^3^ According to Tacla et al.,^20^ patients with low levels of CD4+ T lymphocytes present higher risk of developing CIN, probably due to greater intracellular viral replication. However, we were unable to perform this analysis because no information on CD4+ lymphocyte levels was available from most of the patients. 

## CONCLUSION

The HIV-positive women in our cohort presented a risk of CIN II/III recurrence that was at least 42% higher than among the HIV-negative women. The overall incidence of recurrence was 7.43 per 1,000 women per year, and reached 20.3 per 1,000 women per year if HIV patients alone were considered. Thus, these patients should form part of a more rigorous and more extensive screening and follow-up protocol for identification and appropriate treatment of cervical cancer precursor lesions.
