# ERRATUM

**Published:** 2014-04-01

## ERRATUM

In the article "Recurrence of cervical intraepithelial neoplasia in human immunodeficiency virus-infected women treated by means of electrosurgical excision of the transformation zone (LLETZ) in Rio de Janeiro, Brazil", published in the São Paulo Medical Journal, volume 131, issue number 6, 2013, the correct name of the fourth author is Beatriz Grinsztejn and not Beatriz Gilda Jegerhorn Grinstejn. The article should be cited thus: Russomano F, Paz BR, Camargo MJ, Grinsztejn B, Friedman RK, Tristao MAP, Oliveira CA. Recurrence of cervical intraepithelial neoplasia in human immunodeficiency virus-infected women treated by means of electrosurgical excision of the transformation zone (LLETZ) in Rio de Janeiro, Brazil. Sao Paulo Med J. 2013; 131(6):405-10. PubMed PMID: 24346780. DOI: 10.1590/1516-3180.2013.1316578.



